# NSD2 is a conserved driver of metastatic prostate cancer progression

**DOI:** 10.1038/s41467-018-07511-4

**Published:** 2018-12-05

**Authors:** Alvaro Aytes, Arianna Giacobbe, Antonina Mitrofanova, Katia Ruggero, Joanna Cyrta, Juan Arriaga, Luis Palomero, Sonia Farran-Matas, Mark A. Rubin, Michael M. Shen, Andrea Califano, Cory Abate-Shen

**Affiliations:** 1 Department of Urology, Columbia University Irving Medical Center, 160 Fort Washington Ave, New York, NY 10032 USA; 2 Programs of Molecular Mechanisms and Experimental Therapeutics in Oncology (ONCOBell), and Cancer Therapeutics Resistance (ProCURE), Catalan Institute of Oncology, Bellvitge Institute for Biomedical Research, L’Hospitalet de Llobregat, Gran Via de L’Hospitalet, 199, 08908 Barcelona, Spain; 3 Department of Medicine, Columbia University Irving Medical Center, 630W 168th Street, New York, NY 10032 USA; 4 Department of Systems Biology, Columbia University Irving Medical Center, 1130 Saint Nicholas Ave, New York, NY 10032 USA; 5 Department of Health Informatics, Rutgers School of Health Professions, Rutgers, The State University of New Jersey, 65 Bergen Street, Newark, NJ 07101 USA; 6 Department of Pathology and Laboratory Medicine, Weill Cornell Medicine, 1300 York Avenue, New York, NY 10065 USA; 7 Department for BioMedical Research, University of Bern, Murtenstrasse 35, CH-3008 Bern, Switzerland; 8 Department of Genetics and Development, Columbia University Irving Medical Center, 701 West 168th Street, New York, NY 10032 USA; 9 Herbert Irving Comprehensive Cancer Center, Columbia University Irving Medical Center, 1130 Saint Nicholas Ave, New York, NY 10032 USA; 10 Department of Biochemistry and Molecular Biophysics, Columbia University Irving Medical Center, 701 West 168th Street, New York, NY 10032 USA; 11 Department of Pathology and Cell Biology, Columbia University Irving Medical Center, 630W 168th Street, New York, NY 10032 USA

## Abstract

Deciphering cell-intrinsic mechanisms of metastasis progression in vivo is essential to identify novel therapeutic approaches. Here we elucidate cell-intrinsic drivers of metastatic prostate cancer progression through analyses of genetically engineered mouse models (GEMM) and correlative studies of human prostate cancer. Expression profiling of lineage-marked cells from mouse primary tumors and metastases defines a signature of de novo metastatic progression. Cross-species master regulator analyses comparing this mouse signature with a comparable human signature identifies conserved drivers of metastatic progression with demonstrable clinical and functional relevance. In particular, *nuclear receptor binding SET Domain Protein 2* (*NSD2*) is robustly expressed in lethal prostate cancer in humans, while its silencing inhibits metastasis of mouse allografts in vivo. We propose that cross-species analysis can elucidate mechanisms of metastasis progression, thus providing potential additional therapeutic opportunities for treatment of lethal prostate cancer.

## Abstract (web summary)

Identifying cell intrinsic mechanisms promoting metastasis are necessary to develop new cancer therapeutics. Here they do cross-species computational analysis and identify nuclear receptor binding *SET domain Protein 2 (NSD2)* as a driver of prostate cancer metastasis.

## Introduction

Metastasis is a complex process that culminates in the progressive accumulation of molecular alterations of cancer cells, which allow them to escape the confines of the tumor, survive during dissemination, and ultimately reside at distant sites, wherein requisite adaptive changes ensue in their new microenvironment^1,2^. Therefore, it would be most informative to study the biological processes and molecular mechanisms underlying metastatic progression as occur in the context of the whole organism in vivo. However, inherent challenges in accessing primary tumors and their metastases from cancer patients have made it difficult to study de novo metastasis formation. Moreover, most in vivo studies of metastasis have utilized transplantation models wherein cells or tumors are implanted into host organisms, usually immune-deficient ones. While such investigations have advanced our understanding of metastasis mechanisms and have elucidated factors that promote organ tropism^3^, they may not ideally model the cell-intrinsic mechanisms of de novo metastatic progression. Analyses of genetically engineered mouse models (GEMMs) can overcome these obstacles, since they enable access to tumors and their resultant metastases as they arise de novo during cancer progression in the whole organism^4–7^.

Virtually all prostate cancer deaths are due to metastasis, which arises at advanced disease stages and is often resistant to treatment. Indeed, while patients with locally confined disease have highly favorable outcomes (>95%), the 5-year survival for metastatic prostate cancer is less than 30%^8^. Standard treatment for advanced prostate cancer involves androgen deprivation therapy, which is initially effective but ultimately leads to disease recurrence in the form of castration resistant prostate cancer (CRPC), which is highly aggressive and prone to metastasis^9–12^. While second generation anti-androgens, such as enzalutamide and abiraterone acetate, are now being used for treatment of CRPC^10,11^, treatment failure is often associated with progression to even more aggressive subtypes, including neuroendocrine prostate cancer (NEPC)^12–15^. Frequent sites of prostate cancer metastasis are bone and lymph nodes, however, visceral metastasis, such as to lungs and liver, are becoming more prevalent in aggressive variants and associated with increased lethality and poor prognosis^16^.

Several recent studies have identified the landscape of recurrent genomic alterations in prostate tumors and metastases^17–30^. The culmination of these analyses has revealed that metastatic prostate cancer has a significantly higher burden of mutational and somatic copy number alterations compared with primary tumors^20,21,25,27–30^. These include increased frequency of alterations of key oncogenic and tumor suppressor genes such as *AR*, *PTEN*, *TP53*, and *RB1*, and aberrant activation of key signaling pathways such as the PI-3 kinase, FGF receptor, and RAS signaling pathways^5,31,32^. However, functional analyses of causal drivers of metastatic prostate cancer progression have been hindered by the lack of experimental models that enable biological and molecular investigations of de novo metastasis in context of the whole organism.

In the current study, we have investigated metastatic progression in vivo in a GEMM of prostate cancer. Employing lineage tracing to isolate tumor and metastatic cells, we have defined a molecular signature of metastasis progression. Cross-species computational analyses comparing this mouse signature with a comparable human signature of metastatic prostate cancer progression have identified conserved master regulators of metastasis progression that drive these processes. In particular, we have identified *NSD2* as a conserved master regulator of metastatic prostate cancer progression and a robust marker of lethal prostate tumors. Our findings suggest that cross-species investigations based on analyses of de novo metastasis in GEMMs can be broadly used to elucidate mechanisms of metastatic progression and identify potential new therapeutic opportunities for treatment of lethal cancer.

## Results

### A molecular signature of de novo metastasis progression

**Fig. 1:**
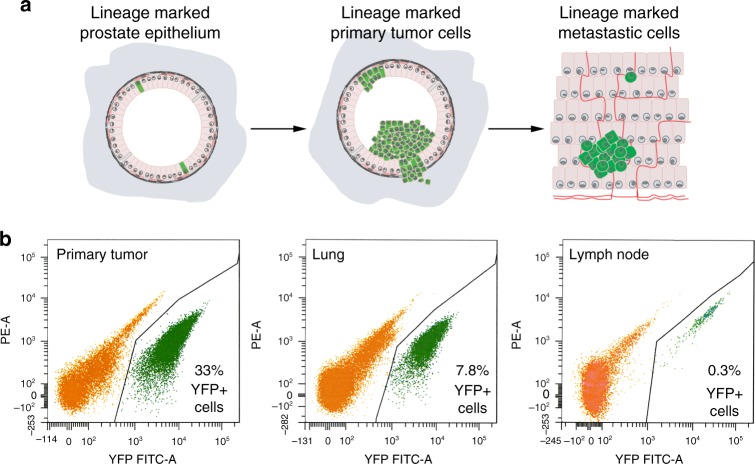
Strategy for molecular profiling of tumors and metastases. **a** Lineage tracing of YFP-labeled (green) prostate epithelial cells at the time of tumor induction leads to YFP-labeled cells in tumors and metastases. **b** Lineage-marked cells from primary tumors, or lung or lymph node metastases were isolated by fluorescence activated cell sorting (FACS). Shown are representative images with percentages of YFP-labeled cells indicated; axes show fluorescent intensity of the fluorescein isothiocynate (FITC-A) and phycoeritrin (PE) channels

To elucidate mechanisms of metastasis progression, we utilized a previously described GEMM of highly penetrant metastatic prostate cancer based on an inducible Cre (Cre^ERT2^) expressed under the control of the promoter of the *Nkx3*.*1* homeobox gene^5^. This *Nkx3*.*1*^*CreERT2*^ allele drives Cre-mediated recombination in an appropriate cell of origin of prostate cancer^33,34^ while simultaneously resulting in heterozygosity for *Nkx3*.*1*, which is prevalent in human prostate cancer^17,27^. We crossed the *Nkx3*.*1*^*CreERT2*^ allele with a *Pten* floxed allele (*Pten*^*flox/flox*^) and an activatable mutant *K-Ras* allele (*Kras*^*LSL-G12D/+*^) to generate *NPK* mice (for *Nkx3*.*1*^*CreERT2*^; *Pten*^*flox/flox*^; *Kras*^*LSL-G12D/+*^). Tumor induction of these *NPK* mice leads to co-activation of *PI3K* and *RAS* signaling, as frequently occurs in lethal prostate cancer in human^5,32^, while these mice develop metastasis with 100% penetrance^5^. These *NPK* mice also contain a conditionally activatable fluorescent reporter allele, *R26R*^*YFP*^, which enables in vivo lineage tracing of primary tumors and their resultant metastases with high efficiency and specificity (Fig. 1a)^5^.

To identify a molecular signature of metastatic progression, we compared expression profiles of primary tumors and metastases from *NPK* mice. Since these mice exhibit temporal progression from pre-invasive (~1 month), to invasive prostate cancer (~3 months), and ultimately to metastasis (~5 months)^5^, we analyzed expression profiles of primary tumors from mice prior to the occurrence of overt metastasis (pre-metastatic, <3 months, *n* = 8), as well as primary tumors from mice that had developed overt metastases (post-metastatic, ~5 months, *n* = 8). Further, since these *NPK* mice metastasize primarily to soft tissues, including lung, liver, and lymph node^5^, we analyzed metastases from these various sites (*n* = 8, 5, 7, respectively); however, since lung is the most prevalent metastatic site^5^, we focused our molecular analyses primarily on lung. As controls, we performed comparable analyses using non-metastatic primary tumors from *NP* mice (for *Nkx3*.*1*^*CreERT2*^*; Pten*^*flox/flox*^; *n* = 7)^35^.

To focus on cell-intrinsic molecular features of primary tumor and metastatic cells free of the surrounding stromal or other components of the microenvironment, which are likely to differ for each tissue, we isolated YFP-lineage-traced cells from tumors and metastases using fluorescence activated cell sorting (FACS) (Fig. 1b). We then performed RNA sequencing on the purified YFP-labeled cells to generate expression profiles corresponding to pre- or post-metastatic primary tumors (*n* = 8/group) as well as lung, liver, and lymph node metastases (*n* = 8, 5, 7, respectively; Supplementary Data 1).

**Fig. 2:**
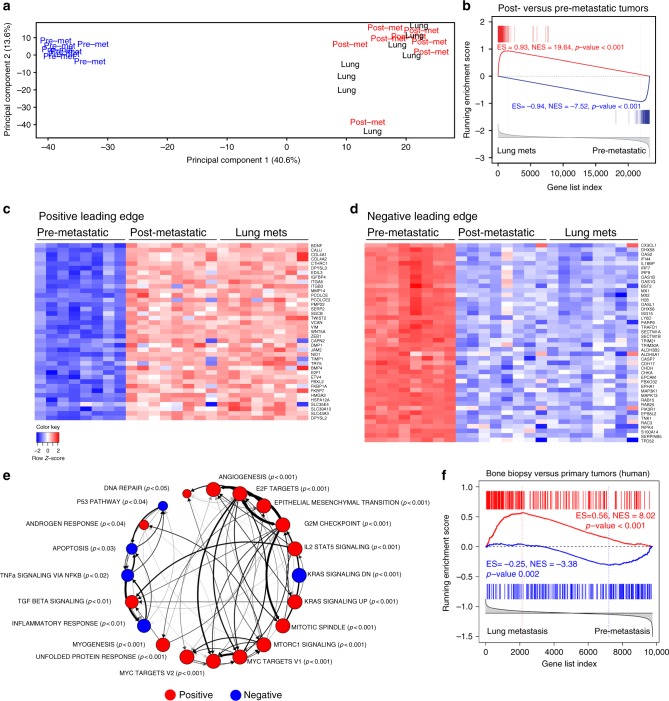
A molecular signature of de novo metastasis progression. **a** Principal component analysis (PCA) comparing expression profiles from pre-metastatic (pre-met, blue) or post-metastatic (post-met, red) primary tumors, or lung metastases (lung, black) from *NPK* mice (*n* = 8/group). Note that Principal Component 1, capturing 40.6% of gene expression variance, effectively distinguishes pre-metastatic tumors from post-metastatic tumors and lung metastases. **b** GSEA comparing a reference signature of mouse lung metastases (lung mets) versus pre-metastatic tumors to a query signature of mouse post-metastatic versus pre-metastatic tumors. **c**, **d** Heat map representations of differentially expressed genes from the positive and negative leading edges, respectively, from the GSEA in panel **b**. The color key shows relative expression levels of the differentially expressed genes (red corresponds to overexpressed genes while blue corresponds to underexpressed genes). **e** Pathway enrichment analysis using the mouse metastasis progression signature defined between lung metastasis versus pre-metastatic tumors, as in panel **b**, to query the hallmark pathways dataset from the molecular signatures database (MSigDB). Red and blue nodes indicate positive and negative enrichment, respectively (*p* < 0.05). Thickness of arrows indicate the overlap of genes in the leading edges. The *p*-values correspond to the GSEA enrichment, and the relative size of the node indicates the relative *p*-value, as shown. **f** Cross-species GSEA comparing a reference mouse metastasis progression signature (lung metastasis versus pre-metastatic tumors, as in panel **b**) with a query gene set from a human metastasis signature defined between bone metastasis biopsies versus primary tumors from Balk et al. (Supplementary Table 1). For GSEA, red vertical bars indicate overexpressed query genes and blue vertical bars indicate underexpressed query genes. GSEA were done using the top 200 differentially expressed genes; *p*-values were calculated using 1000 gene permutations. ES: enrichment score, NES: normalized enrichment score

Interestingly, we found that the expression profiles of the pre- and post-metastatic primary tumor cells were highly dissimilar, whereas expression profiles from the post-metastatic primary tumors were very similar to those from lung, liver, and lymph node metastases (Supplementary Data 1; Fig. 2, Supplementary Fig. 1). Specifically, unsupervised principal component analysis (PCA) revealed that expression profiles from the post-metastatic *NPK* primary tumors clustered more closely with the lung metastases, as well as the metastases to other sites, and further from the pre-metastatic tumors from these *NPK* mice, whereas the pre-metastatic *NPK* tumors tended to cluster more closely with non-metastatic *NP* primary tumors (Fig. 2a; Supplementary Fig. 1a). This relationship was further confirmed by gene set enrichment analyses (GSEA) wherein a differential expression signature comparing post- versus pre-metastatic primary tumors was significantly enriched with a signature of lung metastases versus pre-metastatic tumors in both the positive (NES = 19.64, *p* *<* 0.001) and negative (NES = −7.52, *p* *<* 0.001) leading edges (Fig. 2b). Moreover, expression signatures from the other metastatic sites, namely lung, liver and lymph nodes, were also highly enriched relative to each other (Supplementary Fig. 1b–d), providing further evidence of their similarity.

These observations suggest that the most prominent cell-intrinsic molecular changes that occur during metastatic progression in the *NPK* mice are those that distinguish pre-metastatic from post-metastatic tumors. Hence, taking into consideration: (1) the distinct molecular changes between pre- and post-metastatic *NPK* tumors; (2) the overall similarity of gene signatures of metastatic cells at the various tissue sites (i.e., lung, liver and lymph node); and (3) that the lung is the major metastatic site in the *NPK* mice, our subsequent analyses was done using a signature of metastasis progression based on the differentially expressed genes between the pre-metastatic tumors and lung metastases (*n* = 8/group, respectively; two-sample two-tailed Welch *t*-test; Supplementary Data 1).

Notably, this mouse metastasis progression signature shares molecular features that have been associated with the hallmarks of metastasis progression in other cancer contexts^1,2^. In particular, differentially expressed genes from the positive leading edge of the GSEA (from Fig. 2b) include those associated with epithelial-mesenchymal transition (e.g., *Vim*, *Zeb1*, and *Twist2*), cell and focal adhesion (e.g., *Itga5*, *Col4a1*, and *Col4a2*), membrane type matrix metalloproteinases (e.g., *Mmp14*), and developmental pathways (e.g., *Wnt5A*) as well as other genes known to promote metastasis of prostate (e.g., *Etv4*^5^) or other cancers (e.g., *Hmga2*^7^) (Fig. 2c). Similarly, genes from the negative leading edge (from Fig. 2b) include genes associated with the immune response, such as the interferon regulatory factor *Irf7* which has been shown to be a critical regulator of immunosurveillance in cancer metastasis^36^ (Fig. 2d).

Furthermore, GSEA of biological pathways comparing the mouse metastasis progression signature with the MSigDB Hallmarks dataset revealed a significant enrichment of pathways that are commonly associated with metastatic progression in other tumor contexts, including epithelial to mesenchymal transition, E2F targets, Myc targets, TGF beta, and P53 pathway among others (*p* < 0.05; Fig. 2e; Supplementary Data 2). Notably, many of the pathways enriched in this metastasis progression signature based on tumor versus lung metastases from the *NPK* mice were also enriched in analogous signatures based on tumor versus liver or lymph node metastases (*p* < 0.05; Supplementary Fig. 1e, f), further emphasizing the overall similarity of cell-intrinsic molecular programs associated with metastasis progression across these various organ sites.

Most notably, the mouse metastasis progression signature was highly conserved with a corresponding signature of human metastatic prostate cancer progression reported by Balk and colleagues, which compares primary tumors with metastatic bone biopsies^37^ (Supplementary Table 1). In particular, GSEA comparing a “humanized” version of the mouse metastasis progression signature with the Balk human prostate cancer metastasis progression signature demonstrated their significant similarity in both the positive (NES = 8.02, *p* < 0.001) and negative (NES = -3.38, *p* = 0.002) leading edges (Fig. 2f). Furthermore, GSEA comparing a “humanized” version of the mouse post-metastatic versus pre-metastatic progression signature with this human prostate cancer metastasis progression signature also demonstrated strong enrichment in both the positive (NES = 12.12, *p* < 0.001) and negative (NES = −2.67, *p* = 0.0035) leading edges (Supplementary Fig. 1g). Further, the leading edge genes between these GSEA comparisons (*i*.*e*., from Fig. 2f and Supplementary Fig. 1g) were highly similar (overlap of the positive leading edges was 90.5% and the negative leading edge was 80%; *Χ*^2^ *p* < 0.0001). Taken together, these molecular analyses define a cell-intrinsic signature of de novo metastasis progression in the *NPK* mouse model that is highly conserved with metastasis progression of human prostate cancer.

### Conserved master regulators of metastasis progression

**Fig. 3:**
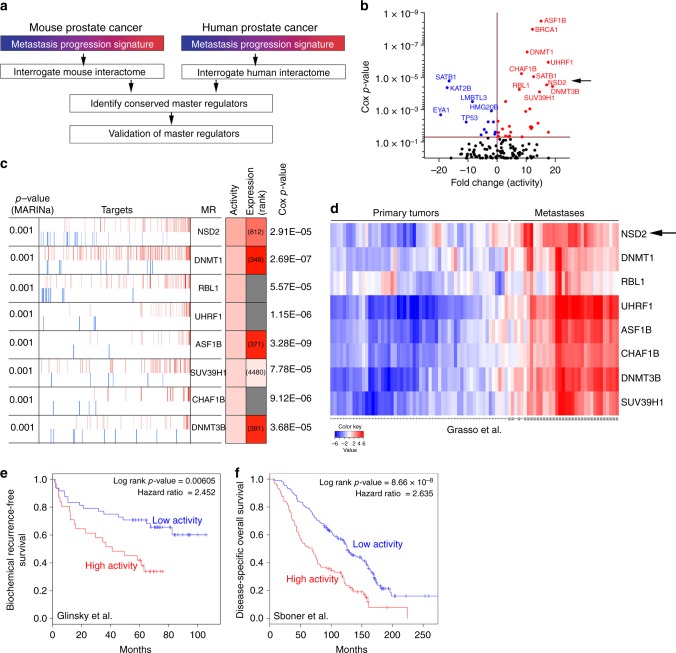
Conserved master regulators of metastasis progression. **a** Strategy: mouse and human metastasis progression signatures (as in Fig. 2f) were used to interrogate mouse and human prostate cancer interactomes, respectively, using the MARINa algorithm. Independent lists of mouse and human master regulators (MRs) were integrated to identify conserved MRs, which were prioritized by clinical and functional validation. **b** Scatter plot showing the association of the 136 conserved MRs (Supplementary Data 3) to clinical outcome using the Sboner et al. dataset, which reports prostate cancer-specific survival as the clinical endpoint (Supplementary Table 1). The *Y* axis represents the Cox proportional hazard *p*-value and the *X* axis represents the fold change based on MR activity. MRs that are inactive (blue) relative to primary tumors have negative fold change values and those that are active (red) have positive fold change values. **c** Summary of the 8 candidate MRs depicting their positive (activated; red bars) and negative (repressed; blue bars) targets. Shaded boxes show the ranks of differential activity and differential expression (darker is higher and lighter is lower); the numbers indicate their rank in the differential expression signature (gray indicates that a specific gene is not present on the utilized gene expression platform, yet its targets are present). *P*-values for Cox proportional hazard were estimated using a Wald test based on time to prostate cancer-specific death in Sboner et al. **d** Heatmap showing hierarchical clustering of primary tumors and metastasis from the Grasso et al. cohort (Supplementary Table 1) based on the activities of the 8 candidate MRs. The color key shows activity levels of MRs (i.e., NESs), where red corresponds to increased activity and blue correspond to decreased activity of the MRs. **e**, **f** Kaplan–Meier survival analysis based on the activity levels of the 8 candidate MRs in: **e** Glinsky et al. (*n* = 79), with biochemical recurrence as the clinical end-point; and **f** Sboner et al. (*n* = 281), with prostate cancer-specific survival as the clinical endpoint (Supplementary Table 1). *P*-values were estimated using a log-rank test to determine the difference in outcomes between patients with higher MR activity levels (red) versus those with lower/no MR activity (blue)

We performed cross-species computational analyses to identify conserved master regulators (MRs) of metastasis progression by interrogating genome-wide regulatory networks, or interactomes, for mouse and human prostate cancer^38^, using the master regulator inference analysis (MARINa) algorithm^39^. First, we interrogated the individual mouse and human prostate cancer interactomes with their respective metastatic progression signatures, which defined independent lists of mouse and human MRs of metastatic progression (Fig. 3a). We subsequently integrated these individual mouse and human MR lists using Stouffer integration to define the subset of conserved candidate MRs (*n* = 485 MRs with Stouffer integrated *p* < 0.0001; Supplementary Data 3). Gene ontology analysis of these integrated MRs revealed an over-representation of genes associated with all aspects of epigenetic regulation, including histone modification, DNA methylation, and chromatin remodeling^40–42^ (*n* = 136/485 genes, 28%; Supplementary Data 3). Because of the potential significance of perturbations of epigenetic regulation for metastatic progression^43,44^, particularly in lethal prostate cancer^21^, and since epigenetic regulators are potential therapeutic targets^40–42^, we focused on the subset of conserved MRs that are predicted to function as epigenetic regulators.

To further prioritize these candidate MRs, we used a Cox proportional hazard model to assess the association of their MR activity with prostate cancer-specific survival (where activity for a given MR is defined based on the expression levels of its MR transcriptional targets, see Methods). In particular, we used a human prostate cancer cohort described by Sboner et al., which has more than 30 years of clinical follow-up data based on death due to prostate cancer^45^ (Supplementary Table 1). These analyses identified a subset of 41 MRs whose activities were significantly associated with prostate cancer-specific survival (Wald test Cox *p* *<* 0.05; Fig. 3b). Among these, we focused our subsequent analysis on 8 candidate MRs: (1) that are associated with adverse disease outcome and prostate-cancer specific lethality (Fig. 3b, c); (2) that are broadly activated in multiple metastatic organ sites (Supplementary Fig. 2a); and (3) whose activities are up-regulated (rather than repressed) in metastasis progression (Fig. 3b, c; Supplementary Fig. 2a), and therefore are potentially targets for treatment inhibition. In particular, these 8 candidate MRs are predicted to be highly activated across multiple metastatic sites, namely lung, liver and lymph node (MARINa *p* < 0.001; Supplementary Fig. 2a), and are significantly associated with adverse disease outcome (Wald test Cox *p* ≤ 10^−5^; Fig. 3c).

We further assessed the clinical relevance of these 8 candidate metastasis MRs using several independent cohorts of advanced prostate cancer patients, including those with clinical endpoints of metastasis or lethality due to prostate cancer (Supplementary Table 1). First, we performed hierarchical clustering on the activity levels of the 8 candidate MRs using the Grasso et al. cohort^46^, which showed that each of these MRs robustly stratify metastases (*n* = 35) from primary tumors (*n* = 59) (Fig. 3d). We observed similar findings with a second cohort that included primary tumors from The Cancer Genome Atlas (TCGA; *n* = 497)^27^ and metastases from the SU2C cohort (*n* = 51)^21^ (Supplementary Fig. 2b).

Additionally, using two independent patient cohorts with clinical follow-up, we found that these 8 candidate MRs predict disease outcome as evidenced by Kaplan–Meier survival analyses based on MR activity levels. In particular, activity of the candidate MRs stratified prostate cancer patients based on their risk of biochemical recurrence using the Glinsky et al. cohort^47^ (*n* = 79 primary prostate tumors; log-rank *p* *=* 0.00605; Hazard ratio = 2.452; Fig. 3e). Furthermore, we found that the activities of these candidate MRs also stratified patients based on the risk of death due to prostate cancer in the Sboner et al. cohort^45^ (*n* = 281 primary tumors; log-rank *p* *=* 8.66 × 10^−8^; Hazard ratio = 2.635; Fig. 3f). Notably, the predictive ability of the 8 candidate MRs for adverse disease outcome in both of these cohorts was highly specific when compared to other MRs selected at random (significance for 8 candidate MRs versus randomly selected MRs was *p*-value = 0.0011 for Sboner et al. and *p*-value *=* 0.0214 for Glinsky et al.; Supplementary Fig. 2c, d).

To evaluate the functional relevance of the 8 candidate MRs for tumor growth and metastasis progression in vivo, we used an allograft cell model derived from the *NPK* mice, which recapitulates the pattern of *NPK* primary tumor growth and metastasis when engrafted into host mice^5^. In particular, we performed shRNA-mediated silencing of each of the candidate MRs using a minimum of 2 shRNAs for each gene (Supplementary Fig. 3a; Supplementary Table 3). Analysis in vitro revealed that *NPK* cells having individually silenced MRs displayed reduced colony formation and reduced invasive potential compared with the control cells, albeit to varying extents for each MR (*p* *<* 0.05, two-tailed Student's *t*-test; Supplementary Fig. 3b–d). Furthermore, when engrafted in vivo, these MR-silenced *NPK* cells displayed reduced tumor growth (*p* *<* 0.05, two-way ANOVA) and/or reduced incidence of metastasis compared with the control cells, also to varying degrees for each MR (*p* *<* 0.05, two-tailed Student's *t*-test; Supplementary Fig. 3e–g). Taken together, these cross-species computational systems analyses have identified conserved master regulators of metastasis progression that are associated with adverse disease outcome and functionally relevant for prostate cancer progression.

### *NSD2* is a driver of metastatic prostate cancer progression

**Fig. 4:**
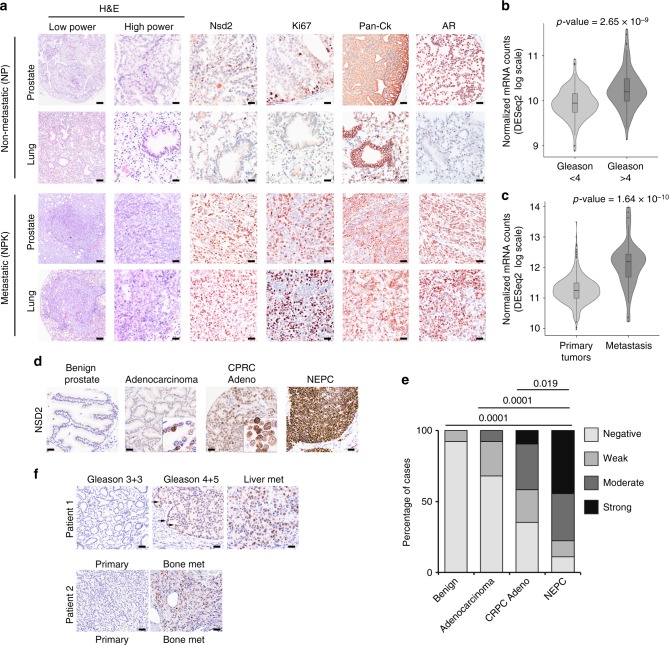
Expression of *NSD2* in prostate cancer and metastases. **a** Immunostaining of Nsd2 and other markers on mouse primary tumors and metastases. Shown are representative H&E images and immunostaining for the indicated antibodies from non-metastatic *NP* mice and metastatic *NPK* mice (*n* = 4/group). Scale bars in the low power H&E images represent 100 microns, and all other panels 50 microns. **b**, **c** Violin plots comparing mRNA expression levels of *NSD2* in TCGA and SU2C human prostate cancer cohorts (Supplementary Table 1). **b** compares primary tumors from TCGA divided based on pathological grade [Gleason <4 (*n* = 104) or ≥ 4 (*n* = 173)], as indicated. **c** compares primary tumors and metastases from a cohort combining primary tumors from TCGA (all Gleason scores; *n* = 333) and metastases from SU2C (*n* = 51). *P*-values were estimated using the two-sample two-tailed Welch *t*-test. **d**, **e** Immunostaining of NSD2 on a human prostate tissue microarray (TMA) (*n* = 100 independent cases). **d** shows representative images representing benign prostate, untreated localized adenocarcinoma, castration-resistant adenocarcinoma (CRPC-Adeno) and neuroendocrine prostate cancer (NEPC). Nuclear staining intensity was evaluated blinded by a pathologist and scored as negative (or present in <5% of nuclei), weak, moderate or strong. Scale bars represent 50 microns. **e** shows quantification of nuclear intensity staining for each score (negative, weak, moderate, and strong). The *p*-values compare negative/weak staining versus moderate/strong staining in each group and were calculated using a two-tailed Fisher's exact test. **f** Immunostaining of NSD2 on matched patient sets of primary prostate cancer and distant metastasis to soft tissues or bone, as indicated. Patient 1 shows representative images of lower pathological grade (Gleason 3 + 3), which is negative for NSD2, and higher pathological grade (Gleason 4 + 5) and a liver metastasis that have increasing expression of NSD2. Patient 2 shows a high grade primary tumor (primary) that is negative for NSD2 and a matched bone metastasis in which NSD2 staining is readily detected. Scale bars represent 50 μ

Among the candidate MRs, the highest level of MR activity (Fig. 3c) as well as experimentally determined functional activity (Supplementary Fig. 3) were observed for the histone methyltransferase, *Nuclear receptor binding SET Domain protein 2* (*NSD2*). Notably, *NSD2* is a putative cofactor of androgen receptor^48^ that has been previously implicated in advanced prostate cancer^49–51^, and has been shown to collaborate with *RAS* signaling in other tumor contexts^52^. Therefore, we examined the expression of *NSD2* at the mRNA and protein levels in non-metastatic and metastatic contexts in both mouse and human prostate cancer (Fig. 4). In the mouse prostate, we found that Nsd2 protein is expressed at low levels in non-metastatic tumors from the *NP* mice, while it is highly expressed in metastatic tumors from the *NPK* mice, as well as corresponding metastases from these mice (*n* = 4/group; Fig. 4a). Notably, Nsd2 was robustly expressed in nuclei of *NPK* tumors and lung metastases, coincident with high levels of Ki67, a marker of cell proliferation, strong expression of nuclear androgen receptor (AR), and robust expression of pan-cytokeratin (Pan-Ck) (Fig. 4a).

In human prostate cancer, we found that *NSD2* expression is increased during cancer progression at both the mRNA and protein levels (Fig. 4b–e). In particular, expression of *NSD2* mRNA levels were significantly higher in more advanced (Gleason ≥ 4 + 4; *n* = 104) versus earlier stage (Gleason <4 + 4; *n* = 173) prostate primary tumors reported in TCGA^27^ (*p* = 2.65 × 10^−9^ two-sample two-tailed Welch *t*-test; Fig. 4b). Further, *NSD2* expression was significantly higher in prostate cancer metastases reported in the SU2C cohort^21^ (*n* = 51) as compared with primary tumors from TCGA (*n* = 333; *p* *=* 1.64 × 10^−10^ two-sample two-tailed Welch *t*-test; Fig. 4c).

To evaluate expression of NSD2 protein in human prostate cancer, we performed immunohistochemistry on a human prostate cancer tissue microarray (*n* = 100) comprised of benign tumors (*n* = 26), non-lethal prostate adenocarcinoma (*n* = 25), lethal castration-resistance adenocarcinomas (CRPC-Adeno; *n* = 31), and neuroendocrine prostate tumors (NEPC; *n* = 18) (Fig. 4d). While NSD2 was either not expressed or expressed at low levels in the non-lethal tumors, its expression increased dramatically in advanced disease stages and was particularly robust in the most aggressive phenotypes, namely CRPC adenocarcinomas and NEPC (*p* *<* 0.01, two-tailed Fisher's exact test; Fig. 4e). To further evaluate the relationship of *NSD2* expression with progression to lethal prostate cancer, we examined matched sets of primary tumors and metastases from the same patient (*n* = 3). Whereas expression in the primary tumors was scattered and focal, NSD2 was robustly expressed in metastasis from these patients (Fig. 4f). These findings extend previous studies showing increased expression of NSD2 in advanced prostate cancer^49^.

**Fig. 5:**
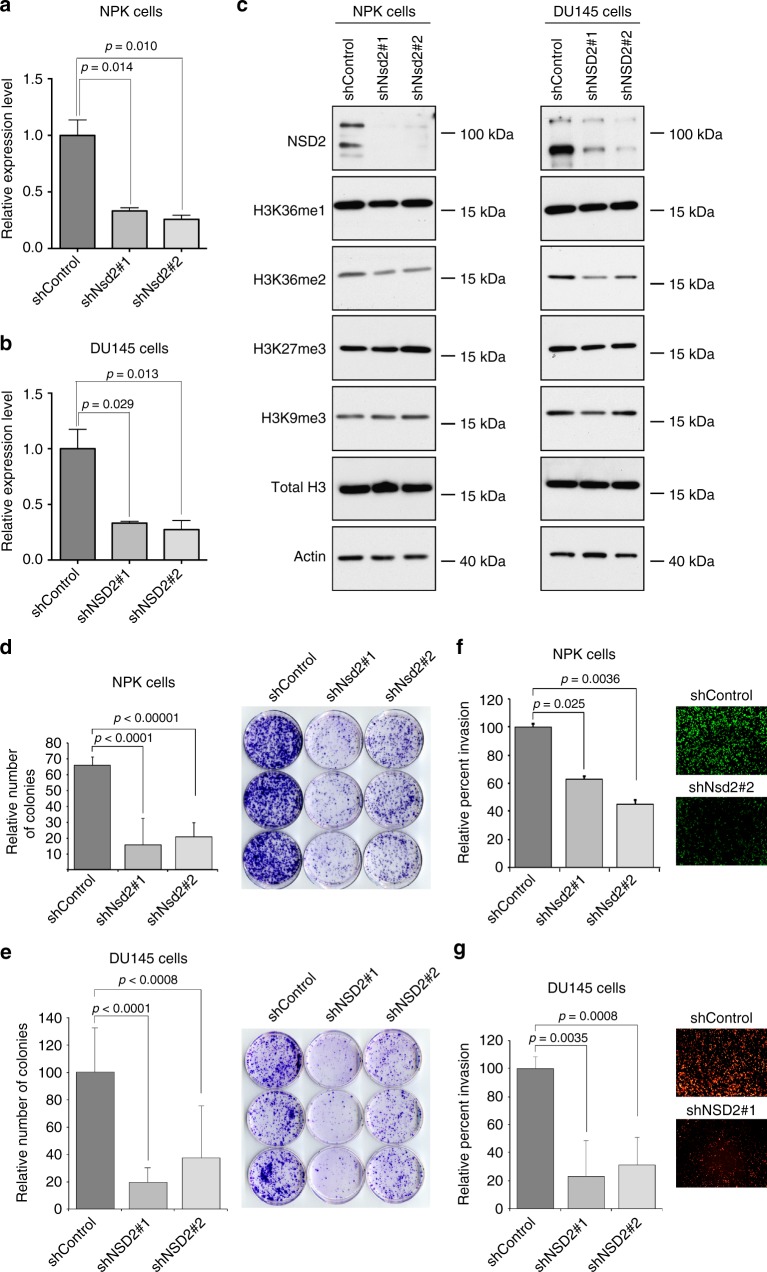
Silencing of *NSD2* abrogates tumorigenicity in vitro. Panels **a**-**g** show in vitro analyses of *NSD2* silencing in a mouse metastatic cell line (*NPK* cells) and a human advanced prostate cancer cell line (DU145 cells). Cells were infected with control shRNA or two independent shRNAs for mouse or human *NSD2*, respectively. **a**, **b** Validation of *NSD2* silencing in *NPK* and *DU145* cells, as indicated, using quantitative real-time PCR (qPCR). **c** Western blot analyses of *NSD2-*silenced or control *NPK* and *DU145* cells, as indicated, showing reduced expression of NSD2, which is accompanied by reduction of the H3K36me2 mark, but not the H3K36m1 or the other histone marks shown. The position of a molecular marker is shown; uncropped images are provided in Supplementary Figure 5. **d**, **e** Colony formation assays in *NPK* and *DU145* cells, as indicated showing quantification (left) representative images (right). **f**, **g** Invasion assays in *NPK* and *DU145* cells, as indicated showing quantification (left) and representative images (right). Experiments were done in three independent biological replicates each in triplicate; *p*-values were calculated using a two-tailed Student's *t*-test. Error bars represent the standard deviation (s.d.) from the mean

To evaluate the functional consequences of *NSD2* for disease progression and metastasis, we used the mouse *NPK* metastatic allograft model, as described above, as well as human DU145 prostate cancer cells, which model aggressive disease^5,38^. In particular, we used lentiviral gene delivery to introduce a minimum of two independent shRNAs to silence *NSD2* in either the mouse or human cells, which resulted in effective silencing of *NSD2* as evident both at the mRNA and protein levels (Fig. 5a–c).

NSD2 has been reported to function as a histone methyltransferase that targets the histone H3 di-methyl mark on lysine 36 (H3K36me2)^51,53–55^. Accordingly, we found that silencing of *NSD2* in either human or mouse cells resulted in a modest but reproducible reduction of the H3K36me2 mark, while not altering the mono-methyl marks on lysine 36 (H3K36me1) or other histone marks such as tri-methyl lysine 27 (H3K27me3) or lysine 9 (H3K9me3) (Fig. 5c). Furthermore, *NSD2* silencing in either mouse *NPK* cells or human DU145 cells in vitro resulted in a 5–10 fold inhibition of colony formation (*p* < 0.0001, two-tailed Student's *t*-test), as well as significantly decreased invasion *(p* < 0.01, two-tailed Student's *t*-test; Fig. 5d–g).

**Fig. 6:**
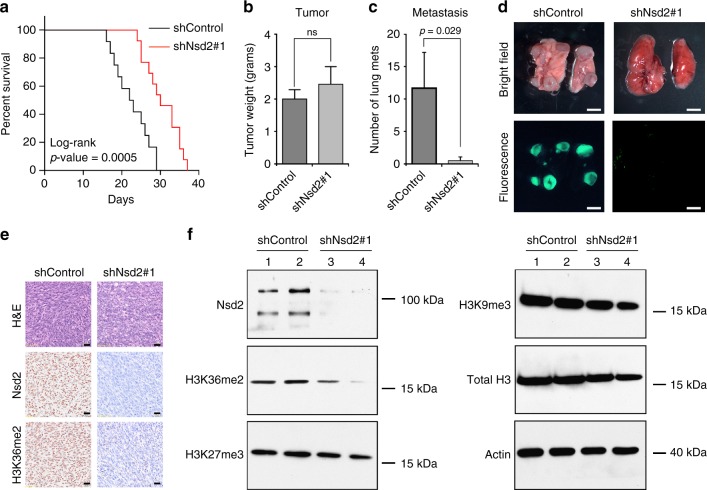
Silencing of *Nsd2* abrogates metastasis in vivo. Panels **a**–**f** show in vivo analyses of *Nsd2* silencing in a mouse metastatic cell line (*NPK* cells). Cells (1 × 10^6^ cells) were engrafted subcutaneously into the flank of *nude* mice and the mice were monitored for up to 40 days. Studies were done using 2 independent shRNA for *Nsd2;* representative data for shRNA#1 is shown. **a** Survival analyses with the endpoint being tumor volume of 1.5 cm^3^ (*n* = 10/group). The *p*-value was calculated using a log-rank test. **b** Analyses of tumor weights (in grams) at the time of killing (total *n* = 9/group). **c** Number of lung metastases per mouse (total *n* = 9/group). **b**, **c** *p-*values were calculated using the Mann–Whitney *U* test; error bars represent the standard deviation (s.d.) from the mean. **d** Representative whole mount and epifluorescence images of lung metastases. Scale bar represent 100 microns. **e** Representative immunostaining of shControl and sh*Nsd2* tumors using the indicated antibodies (*n* = 4/group). Scale bars represent 50 μ. **f** Western blot analysis showing representative cases from the shControl (lanes 1, 2) and sh*Nsd2* (lanes 3, 4) tumors using the indicated antibodies (total *n* = 4/group). The position of a molecular marker is shown; uncropped images are provided in Supplementary Figure 5

Moreover, analyses of *NPK* metastatic allografts in vivo revealed that *Nsd2* silencing resulted in increased overall survival (*n* = 10/group; *p* = 0.0005, log-rank; Fig. 6a) as well as a significant reduction of metastatic burden while not affecting primary tumor growth (*n* = 9/group; *p* *<* 0.03, Mann–Whitney *U* test; Fig. 6b–d). Notably, these *Nsd2*-silenced tumors had profoundly reduced expression of Nsd2 protein compared with the control tumors, as well as reduced expression of the corresponding H3K36me2 mark, but not other histone marks, such as H3K9me3 or H3K27me3 (Fig. 6e, f). Taken together, these observations demonstrate that increased expression of *NSD2* is associated with lethal and metastatic prostate cancer, and establish the functional relevance of *NSD2* for metastatic prostate cancer progression.

**Fig. 7:**
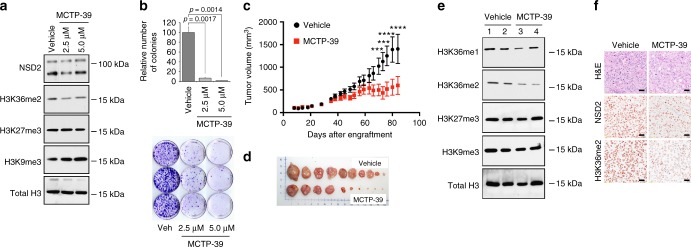
Pharmacological treatment. **a**, **b** Pharmacological treatment in vitro. DU145 cells were treated with MCTP-39 at the indicated concentrations for 72 h. Panel a shows western blot data using the indicated antibodies. The position of a molecular marker is shown; uncropped images are provided in Supplementary Figure 6. **b** depicts colony forming assays showing quantification (top) and representative images (bottom). Shown are representative data from 3 independent experiments, each done in triplicate. Error bars represent the standard deviation (s.d.) from the mean; *p*-values were calculated using a two-tailed Student's *t*-test. **c**–**f** Pharmacological treatment in vivo. DU145 cells (5 × 10^6^ cells) were engrafted subcutaneously into male nude mouse hosts. After 1 week of growth, the tumor-bearing mice were randomized by cage to the vehicle (black) or MCTP-39 (red) treatment groups and treated with 10 mg/kg with MCTP-39 (or vehicle only) for up to 3 months. Tumor volume was monitored using calipers and calculated using the formula [Volume = (width)^2^ x length/2]. Total mice analyzed for vehicle were 14 and for MCTP-39-treatment were 15 in two independent experiments. **c** Tumor volume. Two-way analysis of variance (ANOVA) was used to calculate the significance (*p*-value) of the difference between the vehicle and treatment group; ***p *<* 0.001 and ****p < 0.0001. **d** Representative tumors collected at at the time of euthanasia. **e** Western blot showing 2 examples from vehicle (lanes 1, 2) and MCTP-39 (lanes 3, 4) treated tumors using the indicated antibodies (total *n* = 4/group). The position of a molecular marker is shown; uncropped images are provided in Supplementary Figure 6. **f** Representative immunostaining for NSD2 and H3k36me3 from vehicle and MCTP-39 treated mice (*n* = 4/group). Scale bars represent 50 μ

To consider whether it might be feasible to pharmacologically target *NSD2* activity to inhibit prostate cancer progression and tumor growth, we used a small molecule inhibitor of NSD2 called MCTP-39 (3-hydrazinoquinoxaline-2-thiol), which has been reported to be a lysine-HMTase inhibitor that is a competitor of the SAM (Sterile Alpha Motif) domain^56^. We found that MCTP-39 inhibited the H3K36me2 mark, while not affecting other histone marks such as H3K9me3 or H3K27me3 (Fig. 7a); notably, the degree of reduction the H3K36me2 mark following treatment with MCTP-39 in vitro was comparable to the degree that the H3K36me2 mark was reduced following silencing of *NSD2* in vitro (Fig. 5c).

Treatment with MCTP-39 in human DU145 cells in vitro resulted in a significant dose-dependent reduction in colony formation (>10 fold; *p* < 0.01 two-tailed Student's *t*-test; Fig. 7b). Since we found that this inhibitor was well tolerated in vivo (Supplementary Fig. 4), we evaluated the effect of MCTP-39 on tumor growth of human prostate cells in vivo by establishing DU145 xenografts (Fig. 7c–f). We found that DU145 xenografts treated with MCTP-39 had a significant decrease in tumor volume (*n* = 14 vehicle-treated and *n* = 15 MCTP-39-treated; *p* < 0.001, two-way ANOVA; Fig. 7c, d). The resulting MCTP-39 treated tumors had reduced expression of the H3K36me2 mark, but not other histone marks such as H3K9me3 or H3K27me3 (Fig. 7e, f). Together with the results of silencing *NSD2* in vivo, these findings regarding MCTP-39 treatment suggest that *NSD2* may be a target for intervention in advanced prostate cancer.

## Discussion

Our study demonstrates the value of cross-species integration of molecular data from genetically engineered mouse models (GEMM) and human cancer to elucidate cell-intrinsic mechanisms of de novo metastasis progression. Notably, our current study, which identifies conserved drivers of metastatic progression by isolation of lineage-marked cells directly from tumors and metastases from a prostate cancer GEMM, complements and extends previous work that identified mechanisms of lung cancer metastasis using cell lines generated from tumors and metastases of lung cancer GEMM^7^. Among its advantages for our current investigations, the *NPK* mouse model displays a highly penetrant metastatic phenotype with consistent temporal progression, and it incorporates in vivo lineage tracing of the primary tumors and metastases. Thus, by comparing metastasis progression signatures from *NPK* mice with comparable signatures from human prostate cancer, we have identified conserved master regulators (MRs) of metastasis progression that are associated with adverse disease outcome. We propose that the general strategy of integrating molecular analyses of tumors and metastases from relevant GEMMs with cross-species computational analyses of human cancer can be broadly adopted to identify new targets for prevention, detection, and potentially treatment of metastasis progression for other cancer types.

The capability of generating transcriptomic data from lineage-marked tumor and metastatic cells from different organ sites that are free from other stromal and tissue-specific cells, allowed us to elucidate cell-intrinsic gene expression changes that occur during cancer progression. Surprisingly, we found that the predominant gene expression differences that occur during metastasis progression arise in the transition from pre-metastatic to metastatic tumors and are shared among metastases from various organs. This is similar to findings of a recent study of metastasis progression in mouse model of pancreatic cancer^6^.

Notably, our analyses of purified tumor and metastatic cells free of other tissue components reveals an overall similarity of cell-intrinsic metastasis progression across the various metastatic sites, thus supporting the concept that organ-site tropic factors may be contributed by the tumor microenvironment at the metastatic site^2^. We speculate that organ-site specific factors act in collaboration with cell-intrinsic drivers of metastasis progression, such as those identified herein. Furthermore, our findings, which suggest that there are common cell-intrinsic drivers of metastasis progression across organ sites in the mouse model, is consistent with a study of human prostate cancer, which reported the inherent similarity of tumors and metastases from the same patient^25^, and therefore support the feasibility of investigating agents that target metastatic progression in advanced prostate cancer patients.

Interestingly, we find that conserved master regulators of metastasis progression are highly enriched for genes that are predicted to function as regulators of the epigenome, including those that modify DNA and histones, or remodel chromatin architecture. Consistent with our findings, genomic sequencing of prostate tumors has identified several mutations in epigenetic genes particularly in advanced prostate cancer^20,21,27^. Furthermore, dysregulation of the epigenome is associated with metastatic progression of human prostate cancer^57,58^.

In particular, we have demonstrated that the *Nuclear receptor binding SET Domain Protein 2* (*NSD2)* is a robust marker of lethal metastatic prostate cancer and a key driver of prostate cancer metastasis, extending previous studies that have reported the relevance *of* NSD2 in prostate cancer^49–51^. *NSD2* was discovered as the overexpressed product of the t(4;14)(p16.3;q32.3) translocation in multiple myeloma, and alternatively named *Multiple Myeloma SET domain containing protein* (*MMSET*), and was identified as a target gene on the 4p16 deletion for the Wolf-Hirschhorn Syndrome, and alternatively called *Wolf-Hirschhorn Syndrome Candidate 1* (*WHSC1*)^53^. Previous studies have shown that genomic alterations occur in other cancer types in addition to multiple myeloma including pediatric leukemia and laryngeal tumors^59,60^. In prostate cancer, *NSD2* has been shown to be up-regulated in advanced tumors coordinating with the activation of PI-3 kinase signaling^49^, and to be a cofactor of androgen receptor^48^.

Notably, the role of *NSD2* in cancer has been shown to be dependent on its activity as a histone methyltransferase for the histone H3 di-methyl K36 (H3K36me2)^50,61,62^. In the current study, we show that MCTP-39, a putative inhibitor of *NSD2*^56^, inhibits prostate tumor growth in vivo. However, several caveats preclude us from drawing the direct conclusion that MCTP-39 is acting to inhibit *NSD2* activity in this context, including the potential activity of unknown metabolites and the potential lack of specificity of MCTP-39 given its relatively simple chemical structure^56^.

Nonetheless, our study demonstrates that *NSD2* is a functional driver of prostate cancer metastasis and suggests that it may be target for treatment of advanced prostate cancer. Notably, the activity of *NSD2* as a histone methyltransferase has been shown to be coordinately regulated by EZH2^51^, a major component of the histone methyltransferase polycomb repressive complex 2 (PRC2), which is also dysregulated in prostate cancer. Additionally, in multiple myeloma, *NSD2* has been shown to be a regulator of DNA damage response that impacts resistance to chemotherapy^62^. These previous studies suggest that combination therapy targeting *NSD2* together with inhibition of PI-3 Kinase, AR, EZH2, and/or DNA repair mechanisms, all of which are themselves targetable and highly relevant for prostate cancer, may prove to be efficacious for treatment of metastatic prostate cancer. We further proposed that these combination treatments can be evaluated in co-clinical assays using the *NPK* mouse model described herein.

## Methods

### Expression profiling of lineage-marked cells

All experiments using animals were performed according to protocols approved by and following all ethical guidelines required by the Institutional Animal Care and Use Committee (IACUC) at Columbia University Irving Medical Center or the Ethics Committee for Animal Research (CEIC) at Bellvitge Biomedical Research Institute. For molecular profiling analyses, lineage-marked cells from primary tumors and/or metastases were collected from *Nkx3*.*1*^*CreERT2/+*^*; Pten*^*floxed/floxed*^*; R26R*^*YFP*^ (*NP*) and *Nkx3*.*1*^*CreERT2/+*^; *Pten*^*floxed/floxed*^; *Kras*^*lsl-G12D/+*^; *R26R*^*YFP*^ (*NPK*) mice, which have been previously published^5,33,35^. Note that inclusion of the lox-stop-lox *R26*^*YFP*^ allows for lineage tracing specifically in prostate cells at the time of tumor induction^5^. These *NP*, and *NPK* mice have been maintained in our laboratory on a predominantly C57BL/6 background. All studies were done using littermates that were genotyped prior to enrollment; mice were randomly enrolled to treatment or control groups and only male mice were used because of the focus on prostate.

Mice were induced to form tumors at 2–3 months of age by administration of tamoxifen (Sigma-Aldrich, Allentown, PA) using 100 mg/kg once daily for 4 consecutive days and monitored for 1 to 9 months, during which time the *NPK* mice develop prostate adenocarcinoma that progresses to overt metastasis^5^. At the time of killing, prostate tumors from *NP* or *NPK* mice, as well as tissues with overt metastases from the *NPK* mice, as detected by ex-vivo fluorescence, were collected in ice cold phosphate-buffered saline (PBS). Tissues were digested in one part of collagenase/hyaluronidase (Stem Cell Technologies, Cambridge, MA) and nine parts of DMEM-F12 and 10% fetal bovine serum (FBS) at 37 °C for 3 h. Samples were pelleted at 350XG in an Eppendorf 5810 R tabletop centrifuge for 5 min at 4 °C, re-suspended in 0.25% trypsin/EDTA (Stem Cell Technologies, Cambridge, MA), and incubated for 1 h on ice. Cells were collected by centrifugation as above, and incubated in a cocktail of pre-warmed dispase (5 mg/ml) plus DNaseI (1 mg/ml) (Stem Cell technologies, Cambridge, MA) for 10 min at 37 °C; after which, cells were filtered through a 40 µm cell strainer, pelleted and re-suspended in 1% PBS/FBS and proceed to the sorter. The YFP-lineage marked cells were purified using a BD FACS Aria II sorter and the YFP + population isolated using the PE/FITC (R-Phycoerytrin/Fluorescein isothiocyanate) channels to gate the YFP + population. Cell pellets were resuspended in 10 μl of Trizol and flash-frozen in liquid nitrogen.

RNA was prepared using a MagMAX-96 total RNA isolation kit (Life technologies). Total RNA was enriched for mRNA using poly-A pull-down; only samples having between 200 ng and 1 μg with an RNA integrity number (RIN) > 8 were used. Library preparation was done using an Illumina TruSeq RNA prep-kit, and the libraries were sequenced using an Illumina HiSeq2500 by multiplexing samples in each lane, which yields targeted number of single-end/paired-end 100 bp reads for each sample, as a fraction of 180 million reads for the whole lane. Raw counts were normalized and the variance was stabilized using DESeq2 package (Bioconductor) in R-studio 0.99.902, R v3.3.0 (The R Foundation for Statistical Computing, ISBN 3-900051-07-0). A complete list of differentially expressed genes is provided in Supplementary Data 1.

### Cross species computational analyses

Differential gene expression signatures were defined as a list of genes ranked by their differential expression between any two phenotypes of interest (e.g., metastases vs primary tumors) estimated using a two-sample two-tailed Welch *t*-test. For cross species analyses, the human gene expression signatures were defined based on published prostate cancer cohorts (Supplementary Table 1) and the mouse gene expression signatures were generated from the RNA sequencing analyses as described above (Supplementary Data 1). For the mouse signatures, a minimum of 5 samples were used for each group as necessary to estimate statistical significance in the two-sample two-tailed Welch *t*-test and GSEA.

For comparison with human genes, mouse genes were mapped to their corresponding human orthologs based on the homoloGene database (NCBI) so that mouse-human comparisons were done using the “humanized” mouse signatures. For gene set enrichment analysis (GSEA) normalized enrichment score (NES) and *p*-values were estimated using 1,000 gene permutations. Pathway enrichment analysis was done using GSEA to query the Hallmark Pathways dataset from the MSigDB (i.e., Molecular Signatures Database) collections available from the Broad Institute, where NES and *p*-value were estimated using 1000 gene permutations (Supplementary Data 2). Master regulator (MR) analysis was performed using the MAster Regulator INference algorithm (MARINa) to query the mouse and human prostate cancer interactomes, respectively, as published previously^38^ (Supplementary Data 3).

### Master regulator activity analyses

Transcriptional activity of master regulators (MRs) was estimated using expression levels of their transcriptional targets and reflects their enrichment in the signature being queried. In particular, targets of a particular MR are used as a query gene set to estimate their enrichment in the reference signature of interest (e.g., metastatic progression signature). If positive targets are overexpressed (i.e., enriched in the overexpressed tail of the reference signature) and/or negative targets are underexpressed (i.e., enriched in the underexpressed tail of the reference signature), such MR is considered active (i.e., its transcriptional activity is positive). If the converse is the case, the MR is repressed (i.e., its transcriptional activity is negative).

The relationship of MR activity levels for clinical outcome was assessed using four independent datasets (Supplementary Table 1): Sboner et al., which reports death due to prostate cancer as the clinical end-point^45^; Grasso et al., which reports metastases versus primary tumors as a binary outcome^46^; Glinsky et al., which reports biochemical recurrence (BCR) as the clinical end-point^47^; and a combined SU2C^21^ and TCGA^27^ cohort (i.e., cohorts were combined on raw count levels and normalized using DESeq2 package), which report castration-resistant metastases and primary prostate tumors as a binary outcome, respectively. Sboner et al. was utilized for Cox proportional hazard model analysis (MR filtering/discovery step) and subsequent confirmatory Kaplan–Meier survival analysis. Glinsky et al. was utilized as an independent validation dataset for Kaplan–Meier survival analysis. Grasso et al. and combined SU2C and TCGA cohort were utilized to evaluate the efficacy of MRs stratification of primary tumor versus metastasis.

### Immunohistochemical analysis

All studies involving human subjects were approved by the Institutional Review Board at Weil Cornell Medical School. Only anonymized tissues were used and patient consent was obtained. The cohort included benign prostate tissue (*n* = 26), untreated localized adenocarcinoma (with a representative range of different Gleason scores) (*n* = 25), castration-resistant adenocarcinoma (CRPC-Adeno) (*n* = 31) and neuroendocrine prostate cancer (NEPC) (*n* = 18). Subtype and grading were assigned as defined by pathology and clinical criteria as described^63^. Immunohistochemistry was performed on formalin-fixed paraffin-embedded sections using a Bond III automated immunostainer and the Bond Polymer Refine detection system (Leica Microsystems, IL, USA). Slides were de-paraffinized and heat-mediated antigen retrieval was performed using the Bond Epitope Retrieval 2 solution at pH9 and incubated with the anti-NSD2 primary antibody (Supplementary Table 2). Nuclear staining intensity was evaluated by a pathologist and scoring was done blinded and defined as negative (or present in <5% of nuclei), weak, moderate, or strong. Immunostaining of mouse prostate tissues and metastases was done as described previously^5,35^. Briefly, 3 μm paraffin sections were deparaffinized in xylene, followed by antigen retrieval in antigen unmasking solution (Vector Labs, Burlingame, CA). Slides were blocked in 10% normal goat serum, then incubated with primary antibody overnight at 4 °C, followed by incubation with secondary antibodies for 1 h. For immunostaining, the signal was enhanced using the Vectastain ABC system and visualized with NovaRed Substrate Kit (Vector Labs). All antibodies used in this study, as well as antibody dilutions, are described in Supplementary Table 2.

### Functional validation studies

Mouse cell lines were isolated from lung metastases from *NPK* mice and their genotype was authenticated as described previously^5^. Human cell lines were purchased from and authenticated by ATCC (American Type Culture Collection). Cells were grown in RPMI media supplemented with 10% FBS (ThermoFisher, Bridgewater, NJ). Only early passage cells were used for all studies herein. Cells were routinely tested to ensure that they are free of myoplasma using the MycoFluor Mycoplasma Detection Kit (Invitrogen^TM^, Carlsbad, CA). For shRNA-mediated silencing, a minimum of two independent shRNA clones were used for each gene using the pLKO.1 lentiviral vector system following manufacturer´s instructions (Sigma-Aldrich, Allentown, PA). The sequences for all mouse and human shRNA used in this study are provided in Supplementary Table 3. As a control, we used a pLKO.1 lentiviral vector with shRNA targeting the Luciferase gene (SHC007, Sigma-Aldrich).

Analysis of RNA expression was done by quantitative real time PCR using the QuantiTect SYBR Green PCR kit (Qiagen, Germantown, MD) using mouse or human *GADPH* as the control^5,35^. Sequences of all primers are provided in Supplementary Table 3.

Western blot analysis was performed using total protein extracts as described^5,35^. Briefly, cells were lysed with 1X radioimmunoprecipitation assay (RIPA) buffer (0.1% SDS, 1% deoxycholate sodium salt, 1.0% Triton-X 100, 0.15 M NaCl, 10 mM Tris-HCl (pH 7.5), 1 mM EDTA supplemented with protease inhibitor cocktail (Roche, 11 836 153 001), 1% phosphatase inhibitor cocktail 3 (Sigma, P0044), and 0.5 % PMSF (Sigma). Protein lysates (5 μg per lane) were resolved by SDS-PAGE and transferred onto a PVDF membrane (GE Healthcare, Amersham), then blocked with PBS-T (phosphate-buffered saline and 0.1 Tween-20) containing 5% non fat dry milk. Incubation with primary antibody was done at 4° overnight, followed by incubation with secondary antibody for 1 h at room temperature. Detection was performed using the ECL Plus Western Blotting Detection Kit (GE Healthcare/Amersham, New York). A list of all antibodies and antibody dilutions is provided in Supplementary Table 2. Uncropped images are provided in Supplementary Figures 5 and 6.

Colony formation and invasion assays were done as described^5^. Briefly, for colony formation, 1 × 10^3^ cells were seeded in triplicate in three independent experiments (aggregate total *n* = 9) in 60-mm plates and grown for 10 days in RPMI-1640 media (Gibco, Bridgewater, NJ) supplemented with 10% FBS (BenchMark^TM^ Gemini Bio-Products, Sacramento, CA). Colonies were visualized by staining with crystal violet and quantified using ImageJ software (National Institute of Health website). For matrigel invasion assays, 2.5 × 10^4^ cells were seeded in three independent experiments (aggregate total *n* = 9) in BD FluoroBlok inserts (BD Biosciences, Billerica, MA) in FBS-free media and media supplemented with 10% FBS was used in the lower chamber as a chemoattractant.

For in vivo assays, 1 × 10^6^ cells were injected subcutaneously into the flank of immunodeficient NCr nude mice (male, Taconic, Rensselaer, NY); we performed two independent experiments each done using 10 mice/group. Tumors were monitored by caliper measurement twice weekly for approximately 4 weeks and tumor volumes were calculated using the formula [Volume = (width)^2^ x length/2]. At the time of euthanasia, tumors were harvested and weighed, and the number of lung metastases was documented ex vivo by visualizing their fluorescence using an Olympus SZX16 microscope equipped with epifluorescence capabilities. The total number of metastatic nodules in the lung was assessed for each mouse and the *p*-value was calculated by comparing the control and each shRNA silences using a two-tailed Student's *t*-test or the Mann–Whitney *U* test.

For analyses of NSD2 inhibition, MCTP-39 (3-hydrazinylquinoxaline-2-thiol) was purchased from Enamine L.t.d. (Ukraine)^56^. For studies performed in mice, the MCTP-39 was further purified at the Organic Synthesis Core Facility (OSCF) at Memorial Sloan Kettering Cancer Center. The MCTP-39 was subjected to silica gel chromatography, microfiltered on a 0.2 μ Teflon membrane and lyophilized. The resulting solid was analyzed by UPLC (ultrahigh pressure liquid chromatography) on reverse phase C18 silica gel in addition to ^1^H and ^13^C NMR (Bruker 600 MHz machine).

For in vitro assays, 3 × 10^5^ DU145 cells were seeded in triplicate in three independent experiments (aggregate total *n* = 9) in a 100 mm dish. 24 h following plating, the compound MCTP-39 (5 mM in DMSO) was added to a final concentration of 2.5 μM or 5 μM and incubated for 72 h; DMSO was used as a vehicle control. Cells were collected and lysed for western blot analysis or were seeded for colony formation assays as described above.

For in vivo studies, 5 × 10^6^ DU145 cells in 50% matrigel were injected subcutaneously into the flanks of nude mice. When tumors reached 200 mm^3^, mice were allocated into the vehicle (1% Carboxymethylcellulose, 0.1% Tween80, 5% DMSO) or MCTP-39 (10 mg/kg in vehicle) groups using cage-based randomization, which were administered by oral gavage daily for 3 months. Tumor volume was measured by calipers twice weekly, and estimated by the formula [Volume = (width)^2^ x length/2]. Total mice analyzed were: vehicle = 14; MCTP-39 = 15 (two independent experiments). Two-way analysis of variance (ANOVA) was used to calculate the significance (*p*-value) of the difference between the vehicle and treatment groups.

### Statistical analyses

The Cox model and Kaplan–Meier analysis were done using the surv and coxph functions from the survcomp R package (Bioconductor). Statistical significance was estimated with Wald test and log-rank test, respectively. For Kaplan–Meier survival analysis, *k*-means clustering was done on the activity levels of the MRs to cluster patients into two groups: one group with increased activity of the candidate MRs and one group with decreased MR activity. To compare the predictive ability of candidate MRs to results at random, we selected a random (equally sized, *n* = 8) group of MRs and utilized their activity levels for Kaplan-Meier survival analysis. This procedure was repeated 1000 times and log-rank *p*-values from all iterations were used to build a Null model. The empirical *p*-value was then estimated as a number of times log-rank *p*-values for a random group of 8 MRs reached or outperformed our original log-rank *p* value for the identified 8 MRs. R-studio 0.99.902, R v3.3.0, were used for statistical calculations and data visualization.

## Electronic supplementary material

Supplementary Information

Description of Additional Supplementary Files

Supplementary Data 1

Supplementary Data 2

Supplementary Data 3

Reporting Summary

## Data Availability

The unique raw and normalized RNAseq files are available at Gene Expression Omnibus (GSE111291). A Reporting Summary for this Article is available as a Supplementary information file. All the other data supporting the findings of this study are available within the Article and its Supplementary Information files or from the corresponding authors upon reasonable request.
