# Supplementary material for: NSD2 is a conserved driver of metastatic prostate cancer progression
Source: Nat Commun. 2018 Dec 5;9:5201. doi: 10.1038/s41467-018-07511-4 (PMC6281610; doi:10.1038/s41467-018-07511-4)
Supplement: Supplementary file 1 — Supplementary Information [file 41467_2018_7511_MOESM1_ESM.pdf]

NSD2 is a conserved driver of metastatic prostate cancer progression

Aytes *et al.*

Supplementary Materials

Supplementary Figures

Supplementary Figure 1: Additional RNA sequencing analysis of mouse tumors and metastases. (Related to Figure 2)

Supplementary Figure 2: Additional computational validation of 8 candidate master regulators. (Related to Figure 3)

Supplementary Figure 3: Functional validation of the 8 candidate master regulators. (Related to Figure 3)

Supplementary Figure 4: Safety and tolerability of MCTP-39. (Related to Figure 7)

Supplementary Figure 5: Uncropped scans of the western blots shown in Figures 5 and 6.

Supplementary Figure 6: Uncropped scans of the western blots shown in Figure 7.

Supplementary Tables

Supplementary Table 1: Description of human datasets used in this study

Supplementary Table 2: List of antibodies used in this study

Supplementary Table 3: List of primers and shRNA used in this study

Provided separately

Supplementary Dataset 1: Differentially expressed genes

Supplementary Dataset 2: Pathway analysis

Supplementary Dataset 3: List of mouse and human master regulators

Supplementary Figure 1

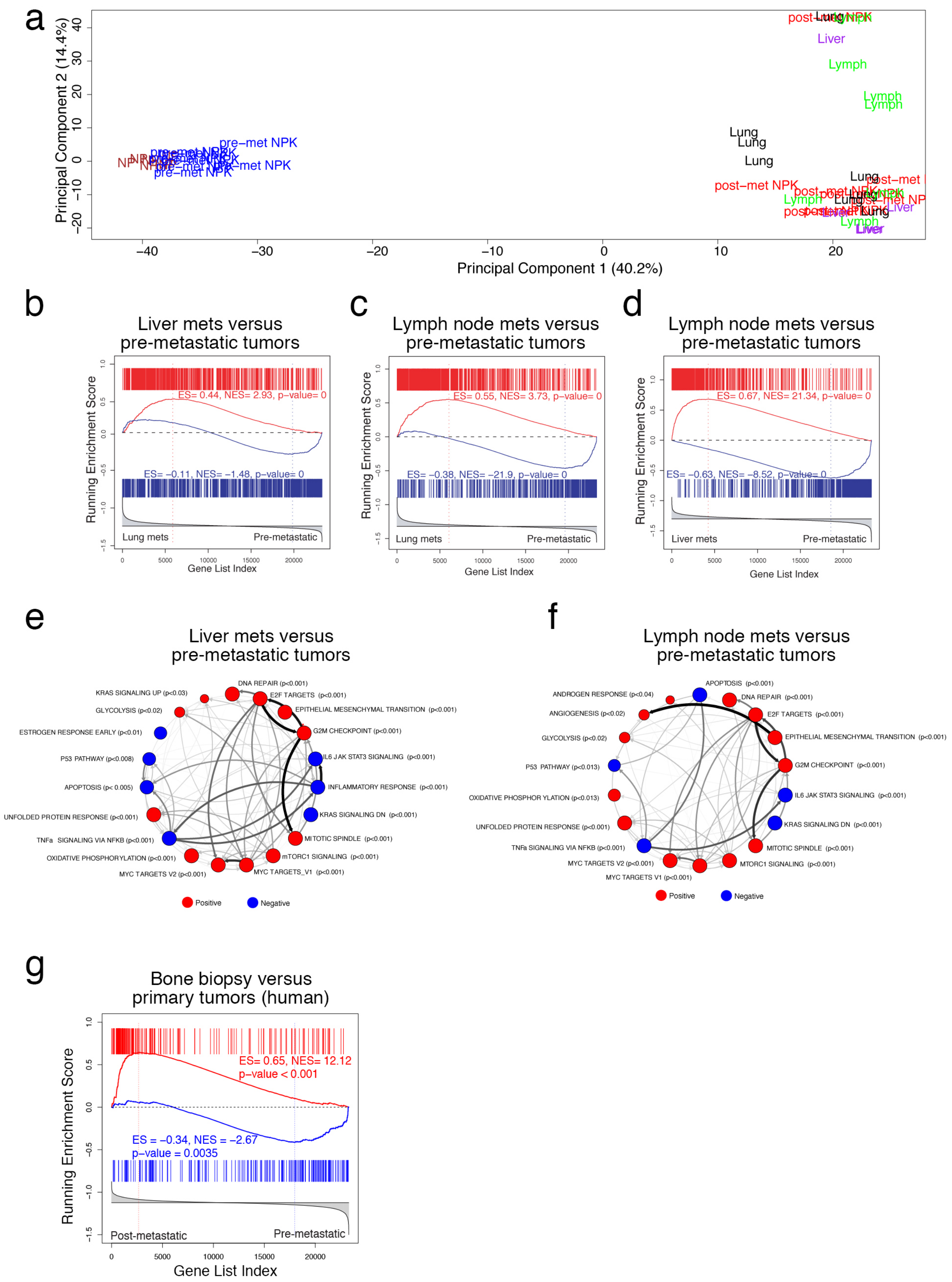

Legend: Additional RNA sequencing analysis. (Related to Figure 2)

(a) Principal Component Analysis (PCA) of RNA sequencing data obtained using YFP-positive FACS sorted cells from: primary tumors from non-metastatic *NP* mice (red), pre-metastatic *NPK* mice (blue), or post-metastatic *NPK* mice (orange), or lung (black), liver (purple), or lymph node (green) metastases from post-metastatic *NPK* mice. Total cases analyzed were: non-metastatic *NP* = 7; pre-metastatic *NPK* = 8; post-metastatic *NPK* = 8; lung mets = 8; liver mets = 5; and lymph node mets = 7.

(b-d) GSEA comparing mouse metastatic signatures. Panels show significant enrichment comparing: (b) the query liver metastasis signature and the reference lung metastasis signature; (c) the query lymph node metastasis signature and the reference lung metastasis signature; and (d) the query lymph node metastatic signature and the reference liver metastasis signature.

(e,f) Pathway enrichment analysis for the liver metastasis signature (e) and lymph node metastasis signature (f). Differential expression signatures were used to query the Molecular Signatures Database (MSigDB) Hallmark pathways dataset. Red and blue nodes indicate positive and negative enrichment, respectively ($p \leqq 0.05$). Thickness of arrows indicate the overlap of genes in the leading edges. The *p*-values correspond to the GSEA enrichment, and the relative size of the node indicates the relative *p*-value, as shown.

(g) GSEA comparing query human metastasis signature with the mouse *NPK* post-metastatic vs *NPK* pre-metastatic reference signature.

For GSEA, red vertical bars indicate overexpressed query genes and blue vertical bars indicate underexpressed query genes. GSEA were done using the top 200 differentially expressed genes; *p* values were calculated using 1,000 gene permutations. ES, Enrichment Score; NES, Normalized Enrichment Score.

Supplementary Figure 2

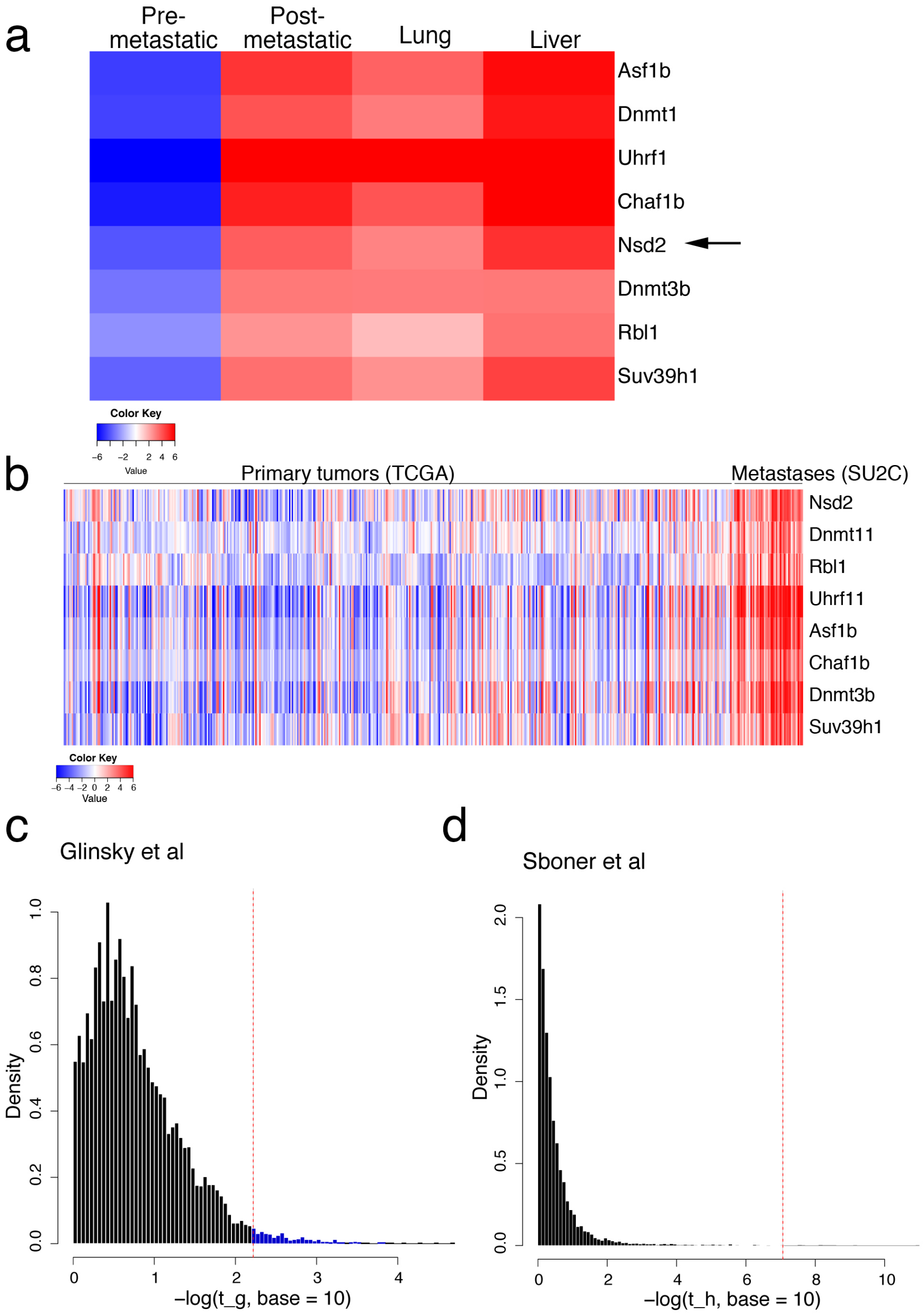

Legend: Additional computational validation of candidate master regulators. (Related to Figure 3)

(a) Heatmap representing master regulator (MR) activity levels of the 8 candidate MRs of metastasis progression (as in Fig. 3c). Shown is the averaged differential activity between pre-

metastatic and post-metastatic *NPK* tumors, and from lung and liver metastases from *NPK* mice. The arrow indicates the Nsd2 activity profile across the indicated tumors and metastases. The color key shows activity levels of MRs (i.e., NESs), where red corresponds to increased activity and blue correspond to decreased activity of the MRs.

(b) Heatmap comparing primary tumors from TCGA dataset (n=51) and metastasis from SU2C datasets (n = 497) (Supplementary Table 1) using the activity levels of the 8 candidate MRs of metastasis progression (as in Fig. 3c).

(c,d) Random models were built and tested on the Glinsky *et al. (c)* and Sboner *et al. (d)* datasets as shown. Density plot depicts the distribution of log-rank *p*-values from Kaplan-Meier survival analysis for 8 MRs chosen at random. Log-rank *p*-value for the candidate 8 MRs is shown as the red vertical lines. Random models were run 1,000 times and a nominal *p*-value was estimated as a number of times log-rank *p*-value of random 8 MRs reached or outperformed log-rank *p*-value for the candidate 8 MRs (blue vertical bars).

Supplementary Figure 3

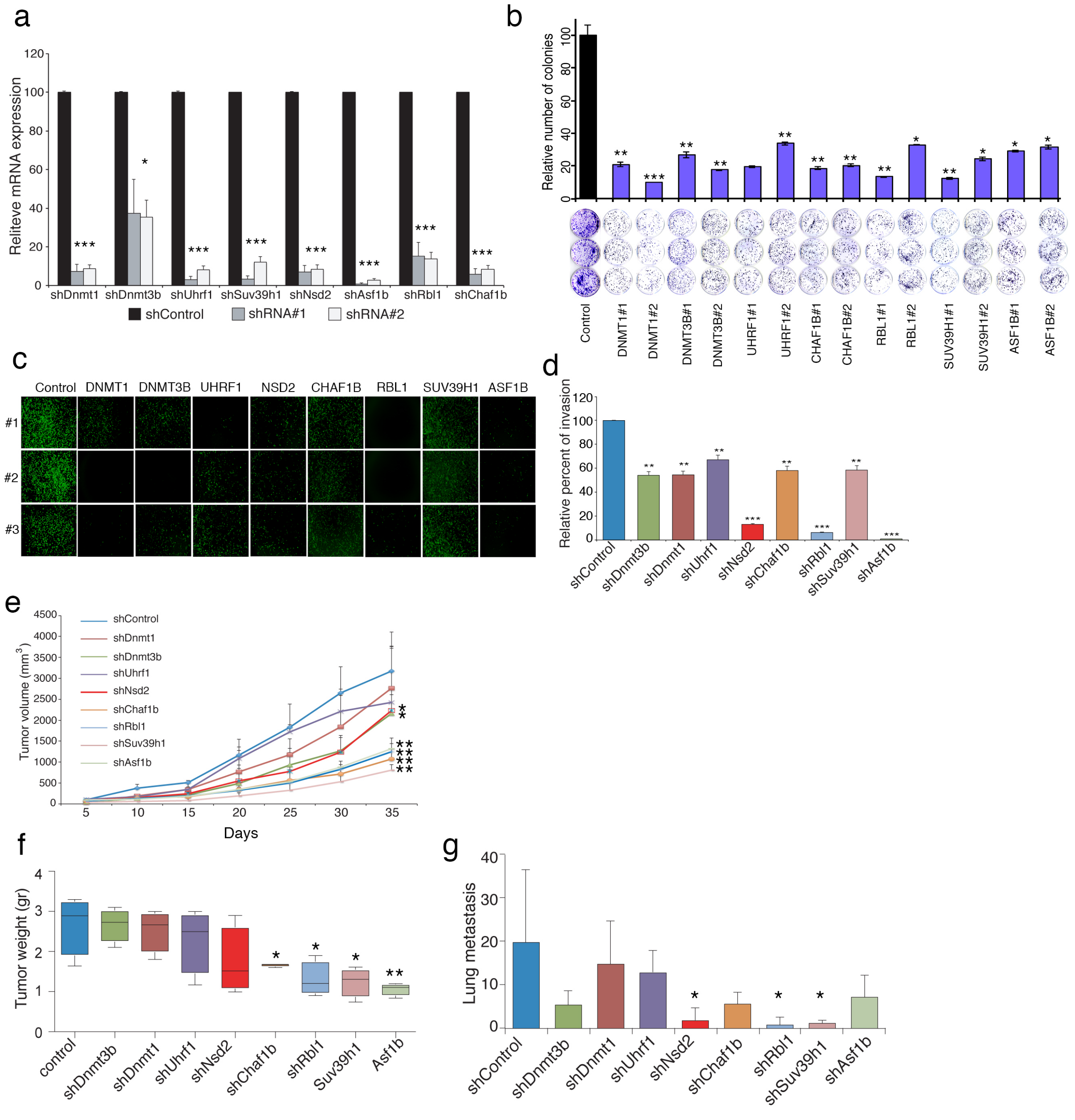

Legend: Functional validation of the 8 candidate master regulators. (Related to Figure 3) For functional validation, *NPK* cells were infected with the control shRNA or two independent shRNAs for each 8 candidate master regulators, as indicated, and assays were done in vitro (a-d) and in vivo (e-g).

(a) Quantitative real-time PCR showing relative mRNA levels of the indicated MRs following shRNA-mediated silencing. Data are normalized to *Gapdh*. Representative data are shown from 3 independent experiments, each done in triplicate. Two-way analysis of variance (ANOVA) was used to calculate the significance (p-value) of the difference between the shControl relative to the targeting shRNA for each individual gene. Error bars represent the standard deviation (s.d.) from the mean.

(b-d) Functional validation in vitro. (b) Colony formation assays showing quantification (top) and representative data (below). (c,d) Invasion assays done using Boyden Chamber assays showing (c) representative microphotographs and (d) quantification.

For (b-d), representative data are shown from 3 independent experiments, each done in triplicate. Two-tailed Student t-test was used to calculate the significance (*p*-value) of the difference between the shControl tumors relative each targeting shRNA. Error bars represent the standard deviation (s.d.) from the mean.

(e-g) Analysis of tumor growth and metastasis in vivo. *NPK* cells silenced with the control shRNA or the shRNAs targeting the indicated candidate MRs were engrafted subcutaneously into the flank of *nude* mice and tumor growth was monitored over time using calipers. Tumors were measured twice weekly for 35 days after allografting and tumor volumes were calculated using the formula [volume = $(\text{width})^2$ x length/2]. (e) Tumor volume over time. Two-way analysis of variance (ANOVA) was used to calculate the significance (*p*-value) of the difference between the control tumors and each of the MR-silenced tumors. (f) Tumor weights taken at the time of

dissection. (g) The number of metastatic nodes in the lungs. The total number of mice analyzed for each candidate MR was 6/group in 2 independent experiments. Statistical differences and *p*-values were calculated using a two-tailed Student t test. Error bars represent the standard deviation (s.d.) from the mean. In all panels, * indicates *p*-value <0.05, ** *p*-value < 0.01, *** *p*-value <0.001 and **** *p*-value <0.0001.

Supplementary Figure 4

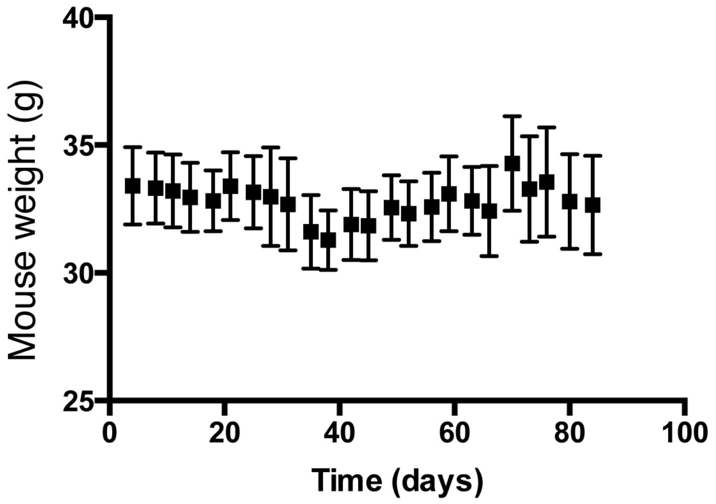

Legend: Safety and tolerability of MCTP-39. (Related to Figure 7)

Safety and tolerability of MCTP-39 assessed in mice by administration of a therapeutic dose of 10mg/kg as in Figure 7c. Body weight was monitored as an indicator of toxicity over 3 months with no significant variations observed.

Supplementary Figure 5

Fig. 5c

Fig. 6f

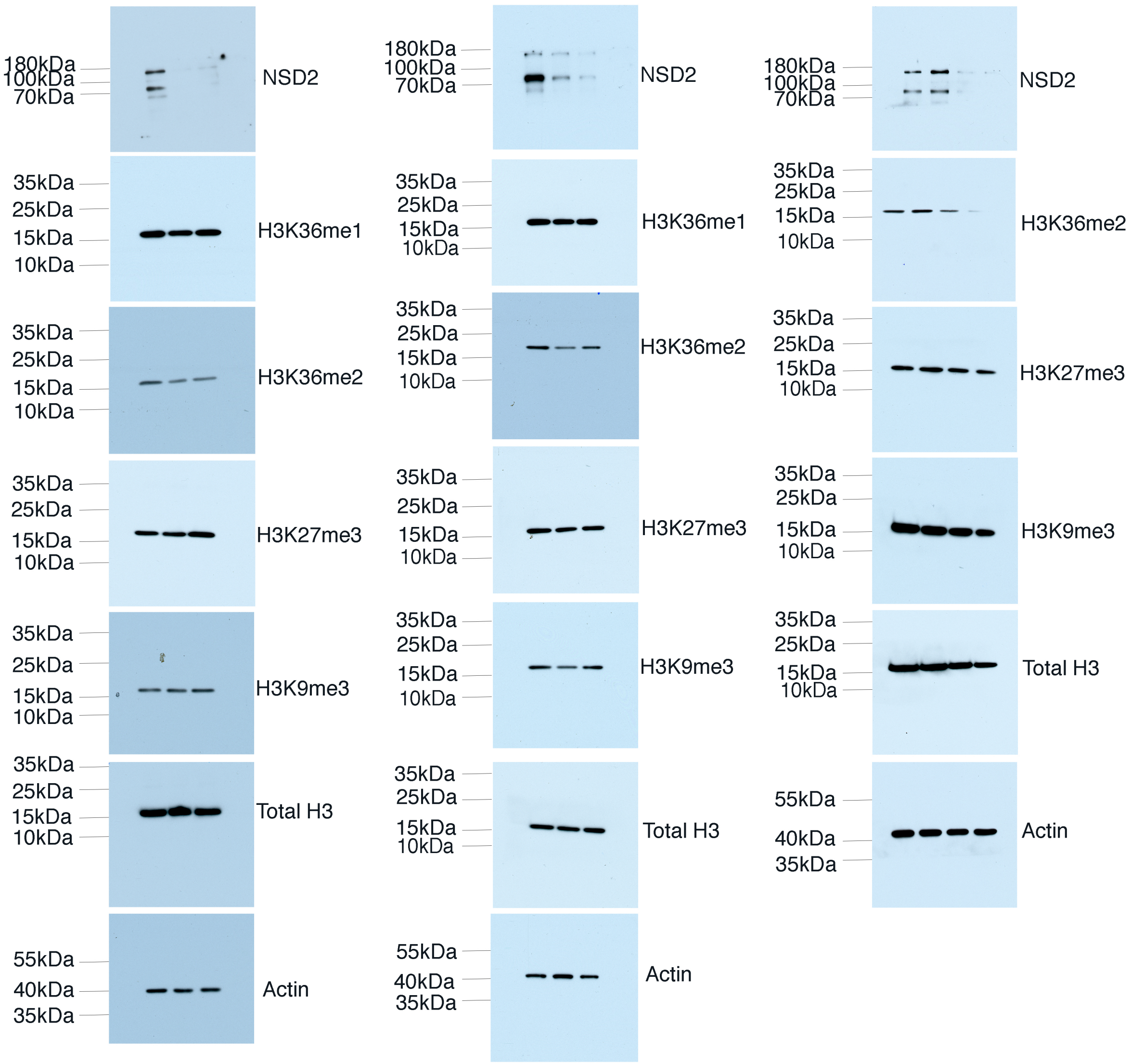

Legend: Uncropped scans of the western blots shown in Figures 5 and 6.

Supplementary Figure 6

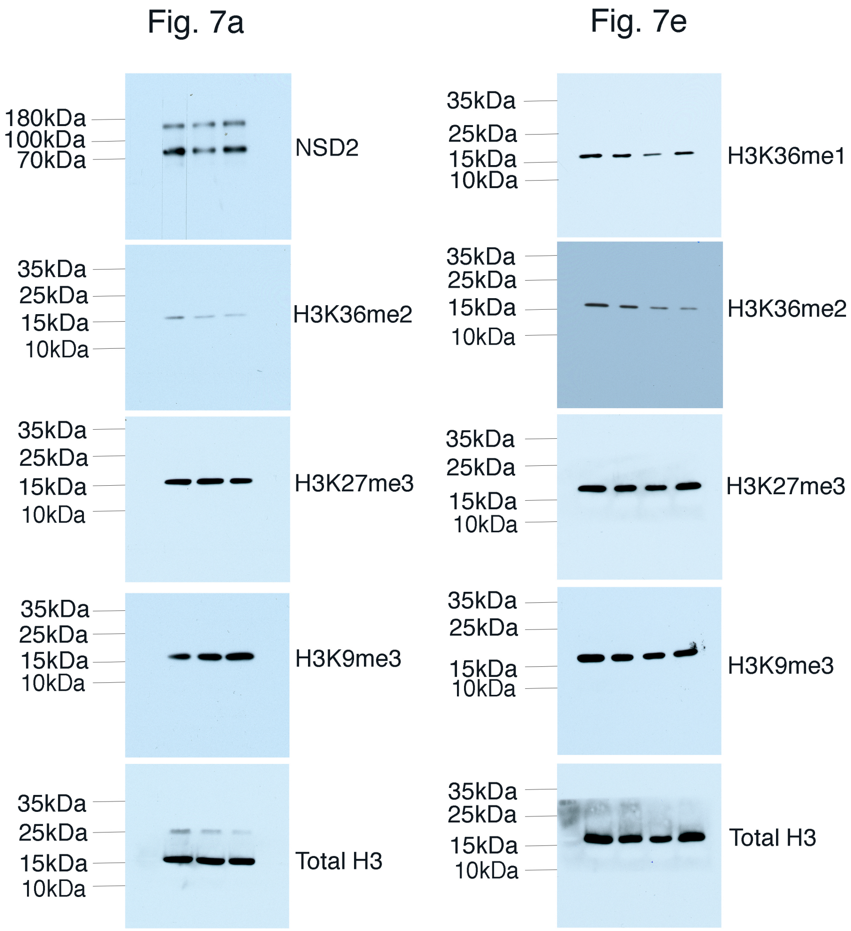

Legend: Uncropped scans of the western blots shown in Figure 7.

Supplementary Table 1: Description of human datasets used in this study				
**Name**	**Description and uses**	**n***	**Platform**	**Geo/Ref**
Balk *et al.*	Description:<br>• Bone metastases from CRPC obtained from bone marrow biopsies<br>• Hormone treatment naïve prostate tumors isolated from frozen biopsies<br>Use:<br>• To generate metastasis signature for MR analysis	29<br>22	Affymetrix Human Genome U133A Array	Geo: GSE32269
Sboner *et al.*	Description:<br>• Transurethral specimens of patients from the Swedish waiting–watchful cohort<br>• One of the few datasets that has extensive clinical follow-up data for a 20-year period including the outcome of death due to prostate cancer.<br>Use:<br>• For clinical validation relative to disease-specific survival	281	Human 6K Transcription-Informative Panel for DASL	Geo: GSE16560
Grasso *et al.*	Description:<br>• Rapid autopsy specimens from metastatic CRPC<br>• Prostatectomy specimens from localized prostate cancer<br>Use:<br>• To stratify metastasis and primary tumors using the 8 MR signature	35<br>59	Agilent Human Genome 44K Array	Geo: GSE35988
Glinsky *et al.*	Description:<br>• Primary prostate tumors from radical prostatectomies in recurrent and non-recurrent patients<br>Use:<br>• For clinical validation relative to biochemical-free survival	79	Affymetrix Human Genome U133A	*NA*
TCGA	Description:<br>• Surgical resection biospecimens from prostate adenocarcinoma without prior treatment<br>Use:<br>• To validate *NSD2* expression in primary tumors	497	Illumina HiSeq 2000 W	TCGA Data Portal
SU2C	Description:<br>• Bone or soft tissue tumor biopsies from metastatic castration-resistant prostate cancer (CRPC)<br>Use:<br>• To validate *NSD2* expression in prostate cancer metastasis	51	Illumina HiSeq 2500	dbGap: phs000915.v1.p1

Supplementary Table 2: List of antibodies used in this study					
**Primary antibodies**					
**Antigen**	**Company**	**Catalog #**	**Type**	**Use and dilution**	
				**IHC**	**Western**
β-actin	Cell Signaling	4970	Rabbit mAb		1:4000
NSD2	Abcam	ab75359	Mouse mAb	1:500	1:2000
AR	Abcam	ab133273	Rabbit mAb	1:200	
H3K36m2	Abcam	ab9049	Rabbit pAb	1:500	1:2000
H3K36me1	Cell Signaling	14111	Rabbit mAb		1:2000
H3K9me3	Abcam	ab8898	Rabbit pAb		1:2000
H3K27me3	Millipore	07-449	Rabbit pAb		1:2000
Histone H3	Cell Signaling	4499	Rabbit mAb		1:2000
Ki67	eBiosciences	14-5698-82	Rat IGa2	1:500	
Pan-Cytokeratin	Dako	Z0622	Rabbit pAb	1:500	

Secondary antibodies				
**Antibody**	**Company**	**Catalog #**	**IHC**	**Western**
Biotinylated anti-mouse IgG	Vector Laboratories	BA-9200	1:300	
Biotinylated anti- rabbit IgG	Vector Laboratories	BA-1000	1:300	
Biotinylated anti-rat IgG	BD Biosciences	559286	1:300	
ECL-anti Mouse IgG	GE Healthcare	NA931V		1:10000
ECL-anti Rabbit IgG	GE Healthcare	NA934V		1:10000

Supplementary Table 3: Description of primers and shRNA used in this study		
**Real Time qPCR**	**Forward**	**Reverse**
**Mouse genes**		
Dnmt1	CGGCTCAAAGACTTGGAAAG	TAGCCAGGTAGCCTTCCTCA
Dnmt3b	CCTGTGGAGTTTCCGGCTAC	GACGCTCTTAGGTGTCACTTC
Uhrf1	CCACACCGTGAACTCTCTGTC	GGCGCACATCATAATCGAAGA
Suv39h1	GAGAGCTTGTCCGACGACAC	CTTCTGCACCAGGTAATTGGC
Nsd2	TGCCAAAAAGGAGTACGTGTG	CTTCGGGAAAGTCCAAGGCAG
Asf1b	CCTTCCGGTTCGAGATCAGC	GGATGGGTTTGGGGCATCAG
Rbl1	AGGGAGAAGTTATACACTGGCT	CCCTTTCCCACAGTAGGAATGA
Chaf1b	TTCACGACGACAGCATGAAGT	TGTCACATTCTCACCAGATTCCA
Gapdh	AGGTCGGTGTGAACGGATTTG	TGTAGACCATGTAGTTGAGGTCA
**Human genes**		
NSD2	TTATTCCAGCCGACAAGCTG	CGCAGTTTGGCATCGTGTG
GAPDH	ACAACTTTGGTATCGTGGAA	GCCATCACGCCACAGTTTC

shRNA	Clone ID	Oligo Seq
shDnmt1# 1	TRCN0000219081	GTACCGGATCTATGGAAGGTGGTATTAACTCGAGTTAATACCACCTTCCATAGATTTTTTTG
shDnmt1# 2	TRCN0000225699	CCGGCTATCGCATCGGTCGGATAAACTCGAGTTTATCCGACCGATGCGATAGTTTTTG
shDnmt3b# 1	TRCN0000071069	CCGGGCTCTGATATTCTAATGCCAACTCGAGTTGGCATTAGAATATCAGAGCTTTTTG
shDnmt3b# 2	TRCN0000071071	CCGGGCACTTTAATCTGGCTACCTTCTCGAGAAGGTAGCCAGATTAAAGTGCTTTTTG
shUhrf1# 1	TRCN0000302343	CCGGCACACACTCTTCGATTATGATCTCGAGATCATAATCGAAGAGTGTGTGTTTTTG
shUhrf # 2	TRCN0000304673	CCGGTCATGTACCATGTCAAGTATGCTCGAGCATACTTGACATGGTACATGATTTTTG
shSuv39h1#1	TRCN0000097439	CCGGGCCTTTGTACTCAGGAAAGAACTCGAGTTCTTTCCTGAGTACAAAGGCTTTTTG
shSuv39h1#2	TRCN0000097441	CCGGCCTGCACAAGTTTGCCTACAACTCGAGTTGTAGGCAAACTTGTGCAGGTTTTTG
shNsd2#1	TRCN0000226297	CCGGAGAGCTGACTTTCAACTATAACTCGAGTTATAGTTGAAAGTCAGCTCTTTTTTG

shNsd2# 2	TRCN0000253039	CCGGCCCACTCCTTCACAATCATACCTCGAGGTATGATTGTGAAGGAGTGGGTTTTTG
shAsf1b# 1	TRCN0000108964	CCGGGTGGGCTACTATGTCAACAATCTCGAGATTGTTGACATAGTAGCCCACTTTTTG
shAsf1b# 2	TRCN0000108961	CCGGCCTCAGTTGCACTCCTGTTAACTCGAGTTAACAGGAGTGCAACTGAGGTTTTTG
shRbl1# 1	TRCN0000218550	GTACCGGATCTTTGCCAATGCTATAATGCTCGAGCATTATAGCATTGGCAAAGATTTTTTTG
shRbl1# 2	TRCN0000234087	CCGGTATCCAATCAGGACCATATAACTCGAGTTATATGGTCCTGATTGGATATTTTTG
shChaf1b #1	TRCN0000092868	CCGGGCTGTCAATGTTGTACGCTTTCTCGAGAAAGCGTACAACATTGACAGCTTTTTG
shChaf1b#2	TRCN0000092871	CCGGTGTGGCTTTCAACATTTCAAACTCGAGTTTGAAATGTTGAAAGCCACATTTTTG
shNSD2# 1	TRCN0000019815	CCGGGCACGCTACAACACCAAGTTTCTCGAGAAACTTGGTGTTGTAGCGTGCTTTTT
shNSD2# 2	TRCN0000019816	CCGGGCACGCTACAACACCAAGTTTCTCGAGAAACTTGGTGTTGTAGCGTGCTTTTT
