# Supplementary material for: NSD2 is a conserved driver of metastatic prostate cancer progression
Source: Nat Commun. 2018 Dec 5;9:5201. doi: 10.1038/s41467-018-07511-4 (PMC6281610; doi:10.1038/s41467-018-07511-4)
Supplement: Supplementary file 2 — Description of Additional Supplementary Files [file 41467_2018_7511_MOESM2_ESM.docx]

**Description of Additional Supplementary Files**

**File Name**: Supplementary Dataset 1: Differentially expressed genes associated with metastatic progression in the NPK mouse model.

**Description**: Differential gene expression comparing: (1A) NPK lung metastases with NPK premetastatic tumors; (1B) NPK post-metastatic tumors to NPK pre-metastatic tumors; (1C) NPK liver metastases with NPK pre-metastatic tumors; and (1D) NPK lymph node metastases with NPK pre-metastatic tumors

**File Nam**e: Supplementary Dataset 2: Biological pathway analysis.

**Description**: Gene set enrichment analysis based on querying the Hallmark pathways of the Molecular Signatures DataBase (MSigDB) showing the enrichment of pathways in: (2A) the lung metastases versus NPK pre-metastatic tumors; (2B) the liver metastases versus NPK premetastatic tumors; and (2C) the lymph node metastases versus NPK pre-metastatic tumors.

**File Name**: Supplementary Dataset 3: List of mouse and human master regulators of metastatic progression.

**Description**: Lists of master regulators of metastatic progression comparing: (3A) human bone metastases with human primary prostate tumors from the Balk et al. dataset interrogated on the human prostate cancer interactome; (3B) the lung metastases versus NPK pre-metastatic tumors interrogated on the human prostate cancer interactome; (3C) conserved master regulators identified by combining the human and mouse master regulators through Stouffer integration; and (3D) the subset of conserved master regulators enriched in the epigenetic gene ontology category.
